# Towards real-time metabolic profiling of a biopsy specimen during a surgical operation by ^1^H high resolution magic angle spinning nuclear magnetic resonance: a case report

**DOI:** 10.1186/1752-1947-6-22

**Published:** 2012-01-18

**Authors:** Martial Piotto, François-Marie Moussallieh, Agnès Neuville, Jean-Pierre Bellocq, Karim Elbayed, Izzie Jacques Namer

**Affiliations:** 1 Bruker BioSpin, 34 Rue de l'Industrie, 67166 Wissembourg, France; 2 Université de Strasbourg, Institut de Chimie, UMR 7177, 4 Rue Blaise Pascal, 67000 Strasbourg, France; 3 Service de Biophysique et Médecine Nucléaire, Hôpitaux Universitaires de Strasbourg, Hôpital de Hautepierre, 1 Avenue Molière, 67098 Strasbourg, France; 4 Service d'Anatomie Pathologique, Hôpitaux Universitaires de Strasbourg, Hôpital de Hautepierre, 1 Avenue Molière, 67098 Strasbourg, France; 5 Université de Strasbourg, Faculté de Médecine, Institut de Physique Biologique, UMR 7237, 4 Rue Kirschleger, 67085 Strasbourg, France

## Abstract

**Introduction:**

Providing information on cancerous tissue samples during a surgical operation can help surgeons delineate the limits of a tumoral invasion more reliably. Here, we describe the use of metabolic profiling of a colon biopsy specimen by high resolution magic angle spinning nuclear magnetic resonance spectroscopy to evaluate tumoral invasion during a simulated surgical operation.

**Case presentation:**

Biopsy specimens (n = 9) originating from the excised right colon of a 66-year-old Caucasian women with an adenocarcinoma were automatically analyzed using a previously built statistical model.

**Conclusions:**

Metabolic profiling results were in full agreement with those of a histopathological analysis. The time-response of the technique is sufficiently fast for it to be used effectively during a real operation (17 min/sample). Metabolic profiling has the potential to become a method to rapidly characterize cancerous biopsies in the operation theater.

## Introduction

**Figure 1:**
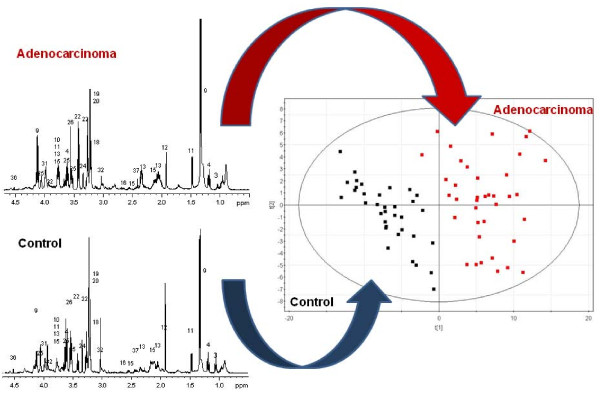
**Statistical partial-least square discriminant analysis model used for the blind test analysis of colon biopsies**. The model was developed and validated using a cohort of 74 colorectal biopsies (control n = 35, adenocarcinoma n = 39) and histopathological criteria using the spectral region corresponding to the taurine, glutamate, aspartate, myo-inositol and glucose resonances. Cancerous and control colon biopsy specimens are represented by red and black boxes respectively. The two component partial-least square discriminant analysis model is characterized by the following parameters: R^2^Y = 0.80 and Q^2 ^= 0.76. The high Q^2 ^value indicates the high predictive capabilities for the model. Representative one-dimensional ^1^H Carr-Purcell-Meiboom-Gill high resolution magic angle spinning nuclear magnetic resonance spectra of control and adenocarcinoma colorectal tissues originating from the same patient are shown on the left side. Partial metabolite assignment in the 4.7 ppm to 0.5 ppm region is indicated. The numbers refer to the following metabolites: (1) isoleucine, (2) leucine, (3) valine, (4) ethanol, (9) lactate, (10) lysine, (11) alanine, (12) acetate, (13) glutamate, (14) methionine, (15) glutamine, (16) aspartic acid, (17) phosphoethanolamine, (18) choline, (19) phosphorylcholine, (20) glycerophosphocholine, (21) arginine, (22) taurine, (23) proline, (24) scyllo-inositol, (25) myo-inositol, (26) glycine, (27) threonine, (28) glycerol, (29) beta-glucose, (30) alpha-glucose, (31) serine, (32) creatine, (33) asparagine, (34) tyrosine, (35) phenylalanine, (36) ascorbic acid and (37) succinic acid.

Providing information on cancerous tissue samples in real-time during a surgical operation can help surgeons delineate more precisely the limits of a tumoral invasion. Currently, only a histopathological examination of the tissue specimen performed under extemporaneous conditions can answer this question reliably. However, this analysis is labor-intensive and its response time can exceed 30 minutes. Metabolic profiling of unprocessed biopsy specimens by ^1^H high resolution magic angle spinning nuclear magnetic resonance (HRMAS NMR) spectroscopy [1-3] has the potential to efficiently distinguish cancerous and healthy tissues [4-10]. However, until now, no attempts have been made to utilize this technique as an additional diagnostic tool in the context of a surgical operation. Recently, a general description of the possibilities offered by this methodology has appeared in the literature [11]. In this paper, a proof of principle of real-time metabolic profiling by ^1^H HRMAS NMR during a surgical operation is given, using as a model the excised colon of a patient with an adenocarcinoma. The metabolic profiles of the different tissue samples of the patient were analyzed, under the time-constraints of a real surgical operation, using a previously established statistical partial-least square discriminant analysis model (R^2^Y = 0.80, Q^2 ^= 0.76) built from 35 healthy colon tissue samples and 39 adenocarcinoma samples. This model is based essentially on the spectral windows corresponding to the chemical shifts of taurine, glutamate, aspartate, myo-inositol and glucose (Figure 1) [12]. The model was used to automatically classify, without human intervention, the new biopsy specimen.

## Case presentation

A 66-year-old Caucasian women presented to our hospital with anemia (hemoglobin 9.1 g/dL, hematocrit 31%) and an abdominal scanner examination revealed the presence of a tumor in her ascending colon. Our patient underwent an open right hemicolectomy with radical lymphadenectomy. The tumor was diagnosed in histopathology as a differentiated adenocarcinoma with angio-, lympho- (2/13) and neuroinvasion and staged as pT4aN1M0 according to the tumor-node-metastasis classification. Molecular analysis of the samples revealed microsatellites-stable tumor with p53 gene alteration. No tumor infiltration was observed in the margin of the resected colon.

## Discussion

Nine biopsy specimens from our patient's segmental colon resection were prepared in 30 μL inserts (preparation time, two minutes per insert) and inserted into a 500 MHz NMR magnet (for detailed experimental procedure see [12]). One-dimensional HRMAS NMR data were then acquired under exactly the same conditions as the ones used to build the statistical model (3°C, rotation speed 3502 Hz, total experiment time 14 minutes). Immediately after data acquisition, the peak integral within each 0.01 ppm region was computed and normalized with respect to the total integral of the spectrum in the 4.7 ppm to 0.5 ppm region using AMIX software (Bruker GmbH, Germany). Datasets were then imported into the SIMCA P 11.0 software (Umetrics AB, Umeå, Sweden), pre-processed using unit variance scaling and input into the previously described statistical model. The model then automatically classified, without any human intervention, the nine samples as either cancerous or control (data analysis time, one minute).

**Figure 2:**
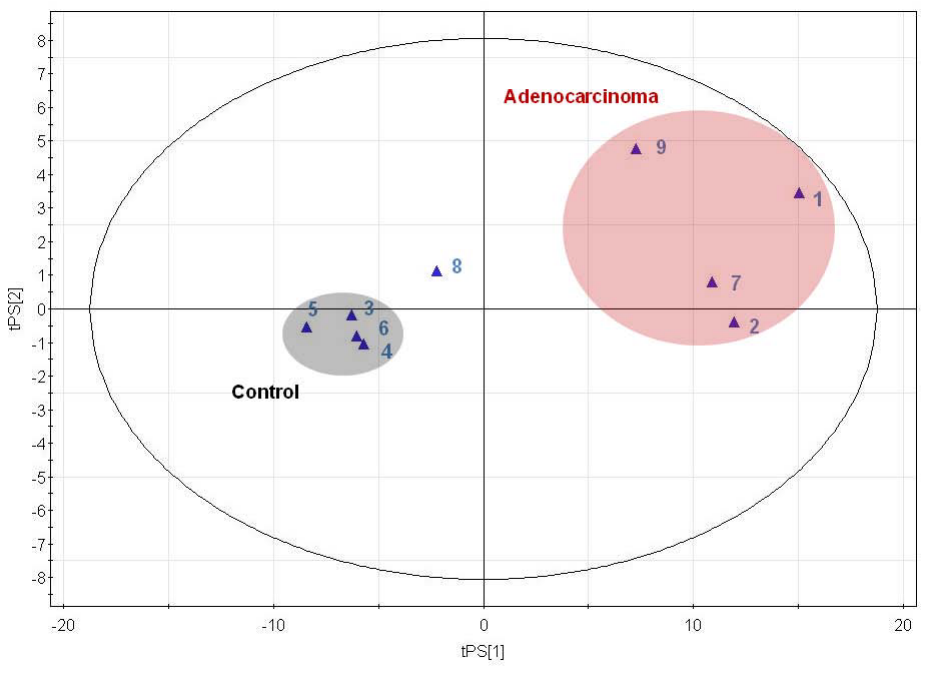
**Blind test classification**. Automatic classification of nine colon biopsy specimens from a patient affected with an adenocarcinoma using the metabolic model presented in Figure 1. Samples 1, 7, 2 and 9 are classified as adenocarcinomas, whereas samples 3, 4, 5 and 6 are classified as control. Sample 8 falls in the border region.

The results of the classification process obtained for our patient are presented in Figure 2. In this score plot, it is observed that one set of four biopsy specimens (biopsies 3, 4, 5 and 6) falls clearly within the control region of the statistical domain, whereas four biopsy specimens (biopsies 1, 2, 7 and 9) appear in the adenocarcinoma region. Biopsy 8 is classified in the border region that separates control samples from adenocarcinomas. A complementary way of analysing the classification data is to check the value of the predicted Y value for each biopsy specimen. In our model, a Y value of 0 corresponds to healthy tissues whereas a Y value of 1 corresponds to an adenocarcinoma. The predicted Y value for biopsy specimens 3, 4, 5 and 6 was found to be 0.15, 0.16, 0.01 and 0.15 respectively whereas the predicted Y value for biopsy specimens 1, 2, 7, 8 and 9 was equal to 1.5, 1.2, 1.2, 0.43 and 1.10 respectively. Clearly, these values reflect the position of each biopsy specimen in Figure 2.

**Figure 3:**
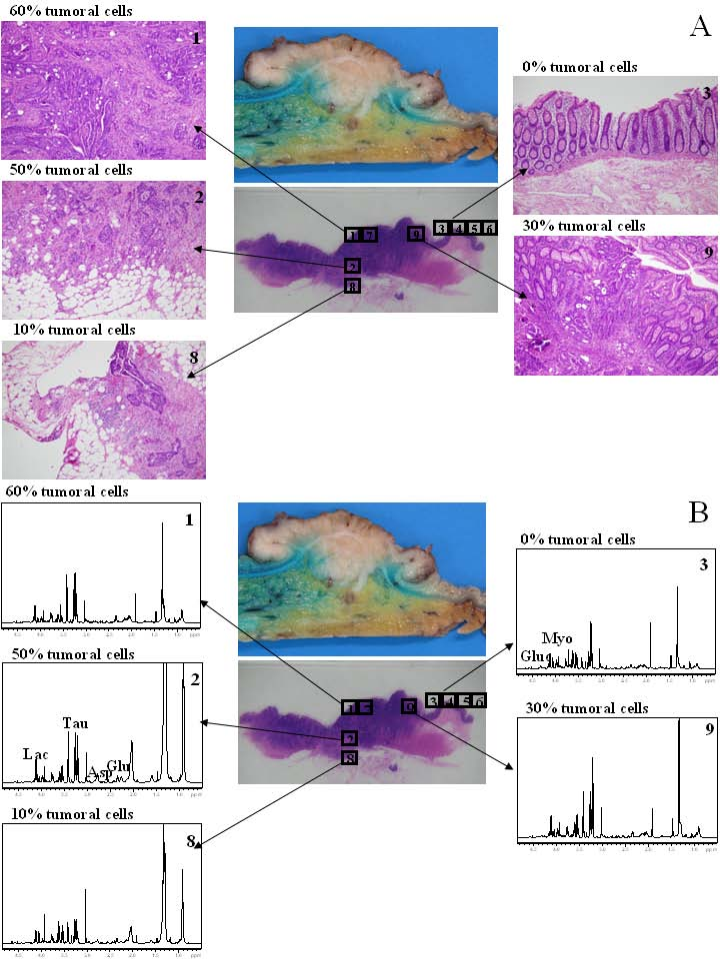
**Combined histopathological analysis and metabolic profiling of colon samples originating from a patient affected with an adenocarcinoma**. A macroscopic view of the colectomy specimen (total length 10 cm) containing an invasive adenocarcinoma with a whitish fungating appearance and a section of the specimen stained with hematoxylin and eosin (H&E). **(A) **Histopathological analysis: The most invasive part of the tumor corresponds to boxes 1, 7 and 9, whereas normal mucosa is represented by boxes 3, 4, 5 and 6. Boxes 2 and 8 correspond to submucosa and serosa tissue respectively. Tissues corresponding to all the nine boxes were embedded in paraffin and stained with H&E for histological analysis (5 μm thickness). Evaluation of H&E staining was performed by light microscopy using a 10× eye-piece with a 20× or a 40× objective. The percentage of tumoral cells indicated was evaluated by a semi-quantitative method and by two pathologists to confirm the diagnosis. **(B) **Metabolic profiling: the histological sections have been replaced by the corresponding one-dimensional ^1^H high resolution magic angle spinning nuclear magnetic resonance spectra. The most representative metabolites of control and adenocarcinoma samples are indicated on the spectra of samples 2 and 3. Samples 2 and 8 contain a higher amount of lipids (large peaks at 0.91 ppm, 1.30 ppm, 1.60 ppm, 2.04 ppm, 2.27 ppm and 2.80 ppm).

A histopathological analysis was performed on mirror samples and on the nine biopsy specimen used for the HRMAS analysis. Quantitative histopathological analysis provided very similar results on mirror samples and on biopsy specimens, despite the fact that some tissue alteration due to rotation was observed. Figure 3 displays a macroscopic view of the colon specimen used with the tumoral zone at the center and the different histopathological sections analyzed. The histopathological analysis revealed that biopsy specimens 1, 7, 2, 9 and 8 contained 60%, 60%, 50%, 30% and 10% of tumoral cells respectively, whereas biopsy specimens 3, 4, 5 and 6, which are located further away from the tumor, contained 0% tumoral cells. When comparing these results with those obtained from metabolic profiling, the results show a remarkable agreement. The results obtained on our patient in Figure 2 show clearly that the position of the different biopsies in the statistical model faithfully reproduce the results of the histopathological analysis. Biopsy specimens 1, 7, 2 and 9 contain the highest number of tumoral cells and are grouped together in the adenocarcinoma region. Control biopsy specimens 3, 4, 5 and 6 correspond to the control region of the statistical model. Biopsy 8 is certainly the most interesting case since it contains only 10% of tumoral cells and the model positions it properly at the border region between control and adenocarcinoma.

Adenocarcinoma are characterized, in decreasing order of importance, by a higher amount of taurine, glutamate and aspartate than control biopsy specimens, which contain more myo-inositol and glucose. Although a detailed analysis of the metabolic pathways affected in ardenocarcinoma cells is out of the scope of this manuscript, it is clear that our results are in full agreement with the Warburg phenomenon [13] since adenocarcinoma cells are clearly depleted in glucose. Figure 3 presents the ^1^H HRMAS NMR spectra corresponding precisely to the biopsy specimens analyzed by histopathology. The higher content of the metabolites taurine (3.42 ppm), glutamate (2.34 ppm) and aspartate (2.80 ppm) is clearly visible in the spectra of tumoral cells, whereas the intensity of myo-inositol (4.05 ppm) and glucose (4.65 ppm) increases significantly in the control biopsy specimens. Thus, the results obtained show that metabolic profiling is indeed able to provide real-time analysis of biopsy samples.

The total time per sample in our case for a full analysis, which consists of sample preparation, data acquisition and statistical analysis, is about 17 minutes per sample. Interestingly, this time is equivalent to that typically observed in the case of an extemporaneous histological analysis but still needs to be reduced in the context of an operation. Realistically, future progress in sample preparation, data acquisition and analysis should be able to reduce this time to less than 10 minutes per biopsy sample. In that respect, automation and simultaneous analysis of multiple samples will play a crucial role for the future development of these techniques.

## Conclusion

Adenocarcinoma of the colon was chosen as a test case for this study for two reasons. First, the segmental colon resection allows the preparation of several biopsies with different levels of tumoral cell infiltration, in particular at the level of the transitional zones. Second, we have previously developed a robust statistical model for this kind of pathology. This model was essential for the success of the current analysis. From a medical point of view, the approach presented here could have some extremely important applications in areas of surgery that require a surgical strategy to be decided under intraoperative conditions, or in cases where it is extremely difficult to delineate with adequate precision the exact zone of tumoral infiltration. Neurosurgery or surgery of the pancreas, in particular, is a field that would benefit immensely from this kind of technology. The prospects of real-time metabolic profiling by HRMAS NMR are therefore extremely promising and methodological as well as technical developments should make this type of analysis even faster.

## Abbreviations

HRMAS: high resolution magic angle spinning; NMR: nuclear magnetic resonance.

## Consent

Written informed consent was obtained from the patient for publication of this case report and any accompanying images. A copy of the written consent is available for review by the Editor-in-Chief of this journal.

## Competing interests

The authors declare that they have no competing interests.

## Authors' contributions

MP, KE and IJN designed the project; FMM and AN performed the research; MP analyzed the data; JPB provided critical assessment and advice on experimental protocols; MP and IJN wrote the paper. All authors read and approved the final manuscript.
